# Development and Validation of a Nomogram for Predicting Severe Respiratory Illness in Children with Isolated Human Metapneumovirus Infection

**DOI:** 10.3390/children13091177

**Published:** 2026-09-01

**Authors:** Dehua Yang, Gang Xiao, Wei Li, Lanfang Tang, Yingshuo Wang, Yunlian Zhou

**Affiliations:** 1 Department of Pulmonology, Children’s Hospital, Zhejiang University School of Medicine, National Clinical Research Center for Children and Adolescents’ Health and Diseases, No. 3333, Binsheng Road, Binjiang District, Hangzhou 310052, China; 2 The First Affiliated Hospital of Ningbo University, No. 59, Liuting Street, Haishu District, Ningbo 315010, China; 3 Department of Clinical Laboratory, Children’s Hospital, Zhejiang University School of Medicine, National Clinical Research Center for Children and Adolescents’ Health and Diseases, No. 3333, Binsheng Road, Binjiang District, Hangzhou 310052, China

**Keywords:** human metapneumovirus, severe pneumonia, children, nomogram, risk factors

## Abstract

**Highlights:**

**What are the main findings?**

• Six independent predictors (sex, neutrophil count, lymphocyte count, prealbumin, crackles, and wheezes) were identified for early prediction of severe respiratory illness in children with isolated hMPV infection.
• The nomogram achieved good discrimination (AUC = 0.743) and calibration, with severe illness rates of 5.1%, 16.7%, and 46.0% across low-, intermediate-, and high-risk groups.

**What are the implications of the main findings? **

• This nomogram enables early risk stratification in hospitalised children within 24 h of admission using routine clinical and laboratory parameters, without relying on chest imaging.
• It provides a practical tool for identifying those at higher risk of progression to severe respiratory illness, facilitating timely monitoring and resource allocation.

**Abstract:**

**Background/Objectives:** Human metapneumovirus (hMPV) is a leading cause of acute respiratory tract infections in children, but comorbidities and mixed infections have confounded most previous studies. This study aimed to develop an early prediction model for severe respiratory illness in children with isolated hMPV infection. **Methods:** This retrospective study enrolled 540 children with isolated hMPV infection (without comorbidities or mixed infections) hospitalised between February 2022 and January 2024. Severe respiratory illness was defined according to British Thoracic Society guidelines. Multivariable logistic regression analysis was performed to identify predictors and develop a nomogram, with model performance evaluated by AUC, Hosmer–Lemeshow test, and bootstrap validation. **Results:** Six independent predictors were identified: male sex (protective factor, OR = 0.500), elevated neutrophil count (OR = 1.114), decreased lymphocyte count (OR = 0.829), decreased prealbumin level (OR = 0.992), crackles (OR = 1.858), and wheezes (OR = 3.734). The nomogram achieved an AUC of 0.743 (95% CI: 0.690–0.796) and demonstrated good calibration performance (Hosmer–Lemeshow *p* = 0.139, Brier score = 0.136). Risk stratification classified patients into low-risk (<10%), intermediate-risk (10–30%), and high-risk (>30%) groups, with severe illness rates of 5.1%, 16.7%, and 46.0%, respectively (*p* < 0.001). **Conclusions:** The nomogram incorporating six early-admission indicators may serve as a practical tool for early risk stratification in hospitalised children with isolated hMPV infection.

## 1. Introduction

Human metapneumovirus (hMPV) is a leading cause of acute respiratory tract infections in children younger than 5 years, accounting for 2.2–10.5% of paediatric respiratory infections and ranking among the most common community-acquired pneumonia pathogens worldwide, along with respiratory syncytial virus (RSV), influenza virus, and rhinovirus [1,2,3]. hMPV infection typically presents with self-limiting upper respiratory symptoms but may progress to severe pneumonia in infants and young children, potentially resulting in respiratory failure and other serious complications [4]. The 2018 Global Burden of Disease study estimated that approximately 643,000 children younger than 5 years are hospitalised annually due to hMPV infection worldwide, with 16,100 deaths attributable to hMPV-associated acute respiratory infections [5]. Therefore, early identification of high-risk children and timely intervention are essential for improving clinical outcomes.

However, most existing studies investigating risk factors for severe hMPV infection have been conducted in real-world, unselected populations and have frequently included children with mixed infections or underlying comorbidities. Fu et al. [6] reported a microbial co-detection rate of 94.8% among hospitalised children with hMPV infection using tNGS and found that polymicrobial co-detection was associated with prolonged hospitalisation and more severe clinical outcomes. Erdoğan et al. [7] identified comorbidities as the strongest independent risk factor for severe respiratory disease in children with hMPV-associated lower respiratory tract infection (OR = 2.96). This is particularly relevant because children with underlying conditions often have a lower threshold for hospital admission, potentially inflating the perceived severity of hMPV infection in unselected cohorts. Excluding such cases allows a more accurate characterisation of isolated hMPV infection. These confounders obscure the specific effects of hMPV, limiting accurate risk assessment in isolated infection. Furthermore, most current prediction models for severe hMPV pneumonia rely on chest imaging or laboratory parameters obtained after hospitalisation, which may hinder early risk stratification. Chest imaging involves risks of radiation exposure and interobserver variability [8,9], whereas laboratory parameters are often obtained after critical clinical decision-making windows. Although the majority of hospitalised children have no underlying comorbidities or mixed infections, a small proportion still progress to severe respiratory illness. Direct evidence regarding the mechanisms through which hMPV alone contributes to severe disease after excluding major confounding factors remains limited.

To address these limitations, the present study retrospectively identified children with isolated hMPV infection among hospitalised cases, determined early warning indicators for severe respiratory illness, and developed a prediction model based on readily available parameters obtainable shortly after admission. This model was intended to facilitate early risk stratification and appropriate referral for hospitalised children.

## 2. Materials and Methods

### 2.1. Study Population

Children hospitalised with positive hMPV test results between February 2022 and January 2024 who fulfilled the eligibility criteria were retrospectively and consecutively enrolled. The inclusion criteria were as follows: age ≤ 18 years; diagnosis of community-acquired pneumonia (CAP); positive hMPV nucleic acid test result; and complete clinical data.

Respiratory specimens were obtained from all children upon admission. Nasopharyngeal swabs were the primary specimen type. For children aged 8 years or older who were able to cooperate, sputum specimens were collected by instructed voluntary coughing and confirmed as qualified by the clinical laboratory before culture. Bronchoalveolar lavage fluid was additionally collected when bronchoscopy was clinically indicated. Data on antibiotic use before admission were extracted from electronic medical records and admission histories, when available. The multiplex RT-PCR assay (RuiSiBei^®^ 13 Respiratory Pathogen Multiplex Detection Kit, Ningbo Health Gene Technologies Co., Ltd., Ningbo, China) was performed within 6 h of admission as part of the standard diagnostic workup.

The exclusion criteria were: (1) underlying comorbidities, including congenital heart disease, asthma (GINA grade ≥ 3), cerebral palsy (GMFCS grade ≥ III), multisystem chromosomal disorders, immunodeficiency, or the use of immunosuppressive agents; and (2) mixed infections, defined as positive nucleic acid test results for bacteria, Mycoplasma pneumoniae, Chlamydia pneumoniae, or other respiratory viruses, or positive bacterial cultures from respiratory specimens or blood. Isolated hMPV infection was defined as a positive hMPV test result with negative results for all aforementioned pathogens.

In China, children may be admitted to hospital through emergency departments, outpatient clinics, or referrals. This study reflects a hospitalised cohort rather than an ED-based population.

### 2.2. Definition of Severe Respiratory Illness and Grouping

Severe respiratory illness was defined according to the 2011 British Thoracic Society (BTS) CAP guidelines [10]. All clinical assessments were performed by attending physicians within 6 h of admission, after the child had been allowed to settle, in the absence of crying or significant distress, and when body temperature was below 37.5 °C where feasible. Patients meeting any of the following criteria were classified as having severe respiratory illness:

(1) Hypoxemia and respiratory distress: cyanosis, tachypnoea (respiratory rate ≥ 70 breaths/min in infants or ≥50 breaths/min in children aged >1 year), signs of increased work of breathing (grunting, nasal flaring, subcostal/intercostal retractions), intermittent apnoea, or oxygen saturation < 92% on room air [10];
(2) Severe chest radiographic abnormalities: chest X-ray or computed tomography (CT) demonstrating consolidation involving ≥1/3 of a lung lobe, bilateral pulmonary involvement, atelectasis, pleural effusion, or pneumothorax.

### 2.3. Data Collection

The following data were extracted from the electronic medical record system: (1) demographic characteristics, including age (years) and sex; (2) physical examination findings, including crackles and wheezes, which were assessed by standardized auscultation within 24 h of admission; (3) laboratory test results, including initial blood test parameters obtained within 24 h of admission, such as white blood cell count (WBC), neutrophil count, lymphocyte count, platelet count (PLT), C-reactive protein (CRP), erythrocyte sedimentation rate (ESR), procalcitonin (PCT), alanine aminotransferase (ALT), aspartate aminotransferase (AST), lactate dehydrogenase (LDH), albumin, and prealbumin; (4) imaging findings, including chest X-ray or CT reports documenting consolidation, the extent of pulmonary involvement (unilateral or bilateral), atelectasis, pleural effusion, or pneumothorax; and (5) treatment and clinical outcomes, including oxygen requirement and duration of oxygen therapy (days). All predictor variables were collected before the determination of the severe illness outcome. The severe illness outcome (including radiographic criteria) was defined as the endpoint event observed during follow-up and was not included as a predictor variable in the model.

### 2.4. Handling of Missing Data

The proportions of missing data for key variables, including age, sex, routine blood parameters, prealbumin, and physical examination findings, were all <1%, and complete-case analysis was performed. ESR and PCT were excluded from the regression analysis because of their relatively high proportions of missing data (approximately 25.0% and 20.4%, respectively). The approach for handling missing data was consistent with the recommendations of the Transparent Reporting of a Multivariable Prediction Model for Individual Prognosis or Diagnosis (TRIPOD) statement.

### 2.5. Statistical Analysis

This study adopted a retrospective cohort design, with severe respiratory illness occurring during follow-up as the outcome and early-admission indicators as predictors. The associations between baseline indicators and subsequent severe outcomes were analysed, and a prediction model was developed. Candidate variables were pre-specified based on previous literature and clinical experience (14 variables in total) and were not modified according to the observed data. Four variables with insufficient supporting evidence from the literature (platelet count, AST, LDH, and albumin) were excluded before analysis. The remaining 10 variables were simultaneously entered into a multivariable logistic regression model using the Enter method. Six variables with *p* < 0.05 were retained in the final model. The linearity assumption for continuous variables was assessed using quartile grouping, and no significant nonlinear deviations were identified. Multicollinearity was evaluated using the variance inflation factor (VIF), with VIF < 5 indicating the absence of significant multicollinearity.

Statistical analyses were performed using SPSS version 22.0 (IBM Corp., Armonk, NY, USA), with a two-sided significance level of α = 0.05. Bootstrap internal validation with optimism correction was conducted using the rms package in R version 4.6.1 (R Foundation for Statistical Computing, Vienna, Austria). Continuous variables were initially assessed for normality using the Shapiro–Wilk test. Normally distributed variables were expressed as mean ± standard deviation and compared between groups using the t-test, whereas non-normally distributed variables were presented as median (IQR) and compared using the Mann–Whitney U test. Categorical variables were presented as n (%) and compared using the χ^2^ test or Fisher’s exact test. Model discrimination was evaluated using the area under the receiver operating characteristic curve (AUC), with an AUC > 0.70 considered acceptable. Model calibration was assessed using the Hosmer–Lemeshow test, with *p* > 0.05 indicating good calibration. A nomogram was developed based on the regression coefficients, and predicted probabilities of <10%, 10–30%, and >30% were defined as low-, intermediate-, and high-risk categories, respectively.

## 3. Results

**Figure 1:**
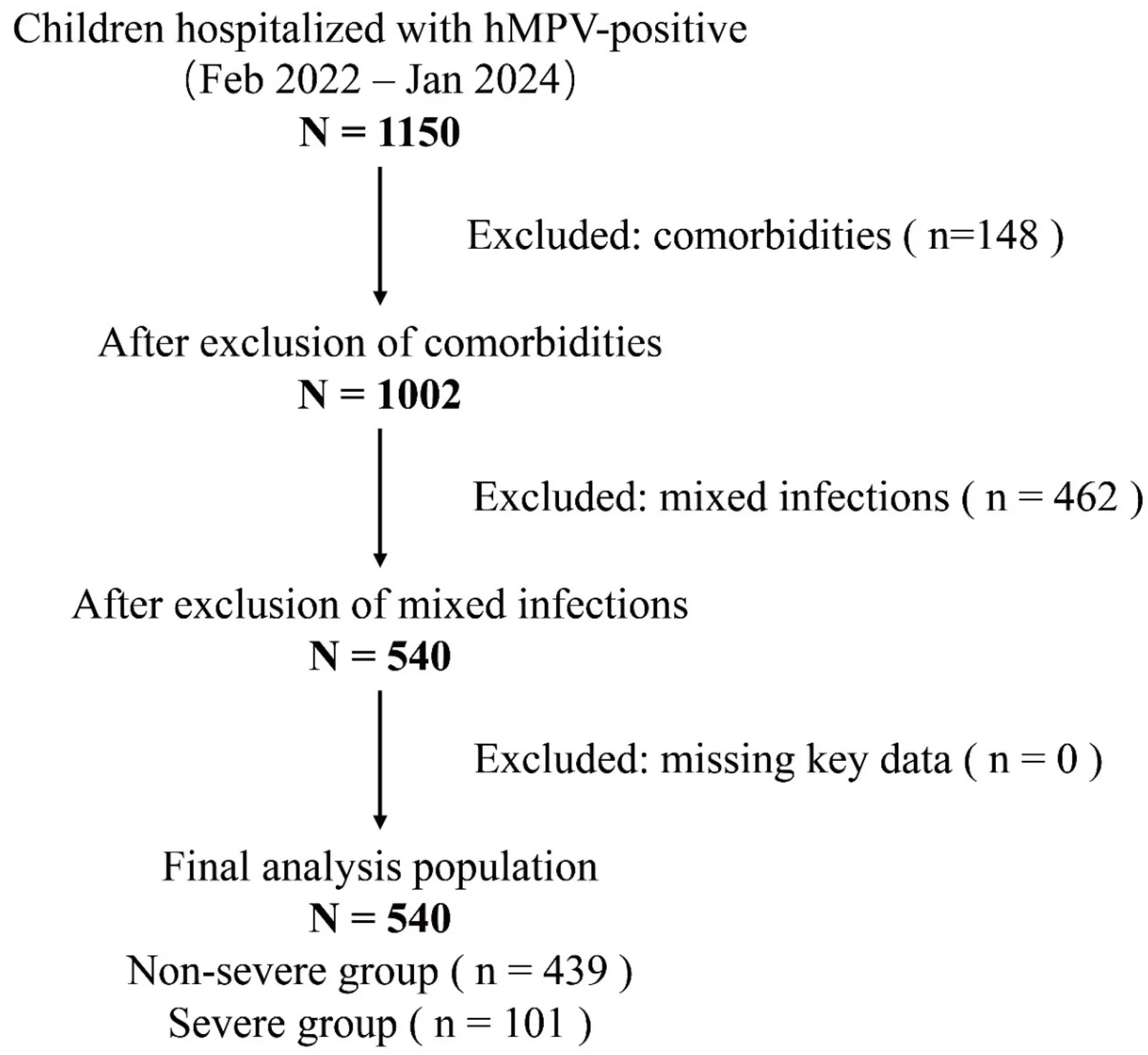
**Study flow diagram of patient selection**. Note: hMPV, human metapneumovirus. The diagram illustrates the screening process from 1150 initial cases to the 540 cases included in the final analysis.

A total of 1150 children hospitalised with positive hMPV test results were initially assessed. After excluding 148 children with comorbidities and 462 children with mixed infections, 540 children with isolated hMPV infection were included in the final analysis (Figure 1). Among these patients, 101 (18.7%) were classified as having severe respiratory illness, whereas 439 (81.3%) were classified as having non-severe illness. Among the 101 children with severe respiratory illness, 77 (76.2%) fulfilled only the hypoxaemia/respiratory distress criteria, 16 (15.8%) fulfilled only the radiographic criteria, and 8 (7.9%) fulfilled both criteria.

Age did not differ significantly (*p* > 0.05); however, the proportion of females was higher in the severe group (*p* = 0.024). The severe group had higher neutrophil, CRP, ESR, and PCT and lower lymphocyte and prealbumin (all *p* < 0.05). Physical examination findings showed that the proportions of crackles and wheezes were significantly higher in the severe group than in the non-severe group (both *p* < 0.05) (Table 1).

Multivariable logistic regression analysis identified sex (male vs. female, OR = 0.500, 95% CI: 0.311–0.805, *p* = 0.004), neutrophil count (per 1 × 10^9^/L increase, OR = 1.114, 95% CI: 1.045–1.189, *p* = 0.001), lymphocyte count (per 1 × 10^9^/L increase, OR = 0.829, 95% CI: 0.730–0.942, *p* = 0.004), prealbumin level (per 1 mg/L increase, OR = 0.992, 95% CI: 0.985–0.999, *p* = 0.017), crackles (present vs. absent, OR = 1.858, 95% CI: 1.159–2.977, *p* = 0.010), and wheezes (present vs. absent, OR = 3.734, 95% CI: 2.240–6.225, *p* < 0.001) as independent predictors of severe respiratory illness in children with isolated hMPV infection (Table 2).

Based on the results of the multivariable logistic regression analysis, the predicted probability (*p*) of severe respiratory illness was calculated using the following formula:

Logit(*p*) = −0.645 + (−0.693 × *Sex*) + (0.108 × *Neutrophil*) + (−0.187 × *Lymphocyte*) + (−0.008 × Prealbumin) + (0.619 × *Crackles*) + (1.318 × *Wheezes*)

*p* = 1/(1 + e^(−Logit))

where Sex was coded as female = 0 and male = 1; crackles and wheezes were coded as absent = 0 and present = 1; neutrophil and lymphocyte counts were expressed as ×10^9^/L; and prealbumin was expressed in mg/L.

**Figure 2:**
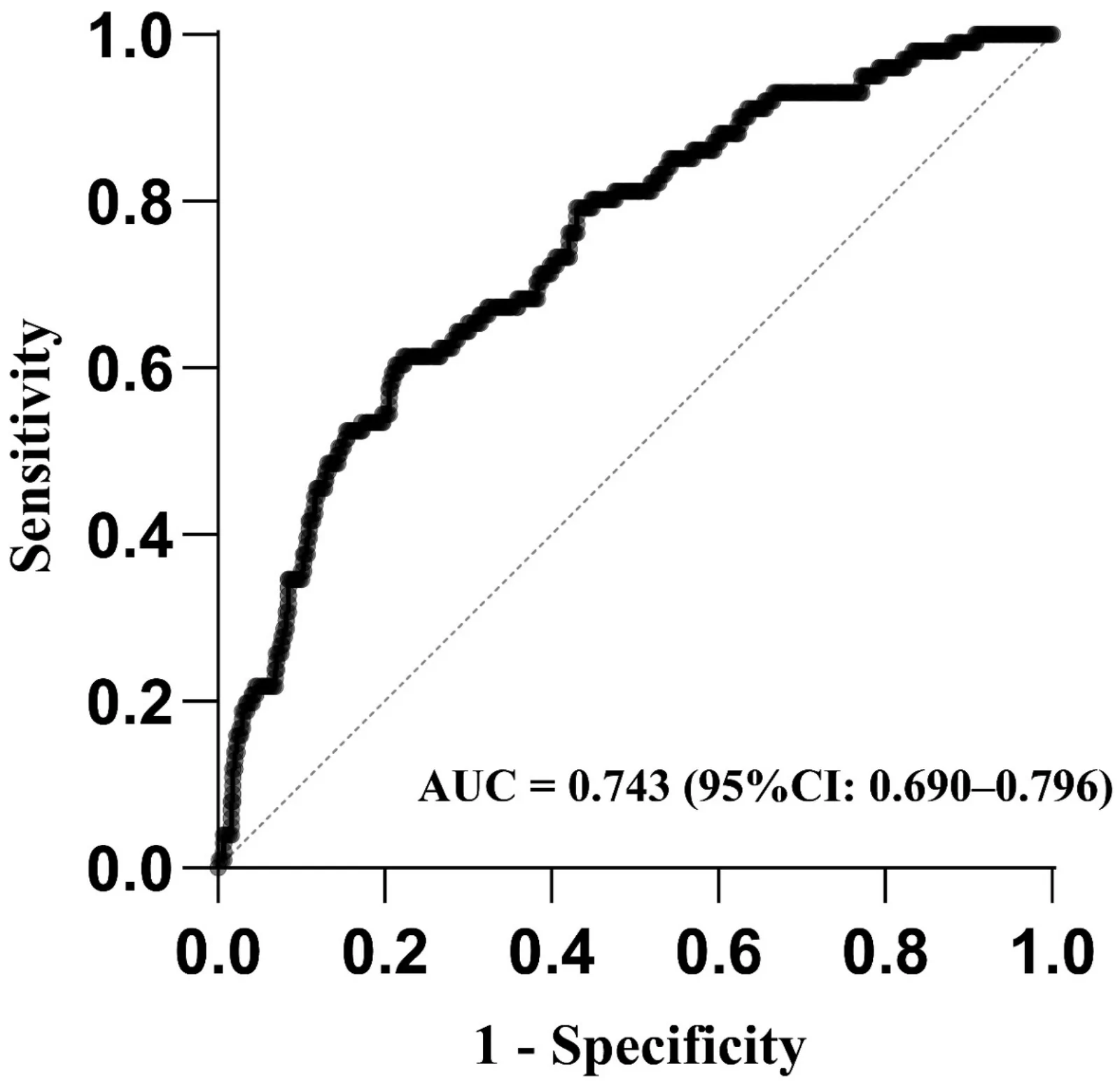
**Receiver operating characteristic (ROC) curve of the prediction model for severe respiratory illness.** Note: ROC, receiver operating characteristic; AUC, area under the curve; CI, confidence interval. The model achieved an AUC of 0.743 (95% CI: 0.690–0.796), indicating moderate discriminative ability. The diagonal dashed line represents the reference line (AUC = 0.5).

ROC curve analysis demonstrated that the model achieved an AUC of 0.743 (95% CI: 0.690–0.796, *p* < 0.001) for predicting severe respiratory illness, and the Hosmer–Lemeshow test indicated good calibration performance (χ^2^ = 12.433, *p* = 0.139) (Figure 2). Bootstrap internal validation (1000 resamples) showed that the BCa 95% confidence intervals for all variables did not cross zero, and the bias values were all < 3% of the original coefficients. The optimism-corrected AUC was 0.744, with a calibration intercept of 0.039, calibration slope of 1.020, and Brier score of 0.136, indicating good model performance and calibration.

**Figure 3:**
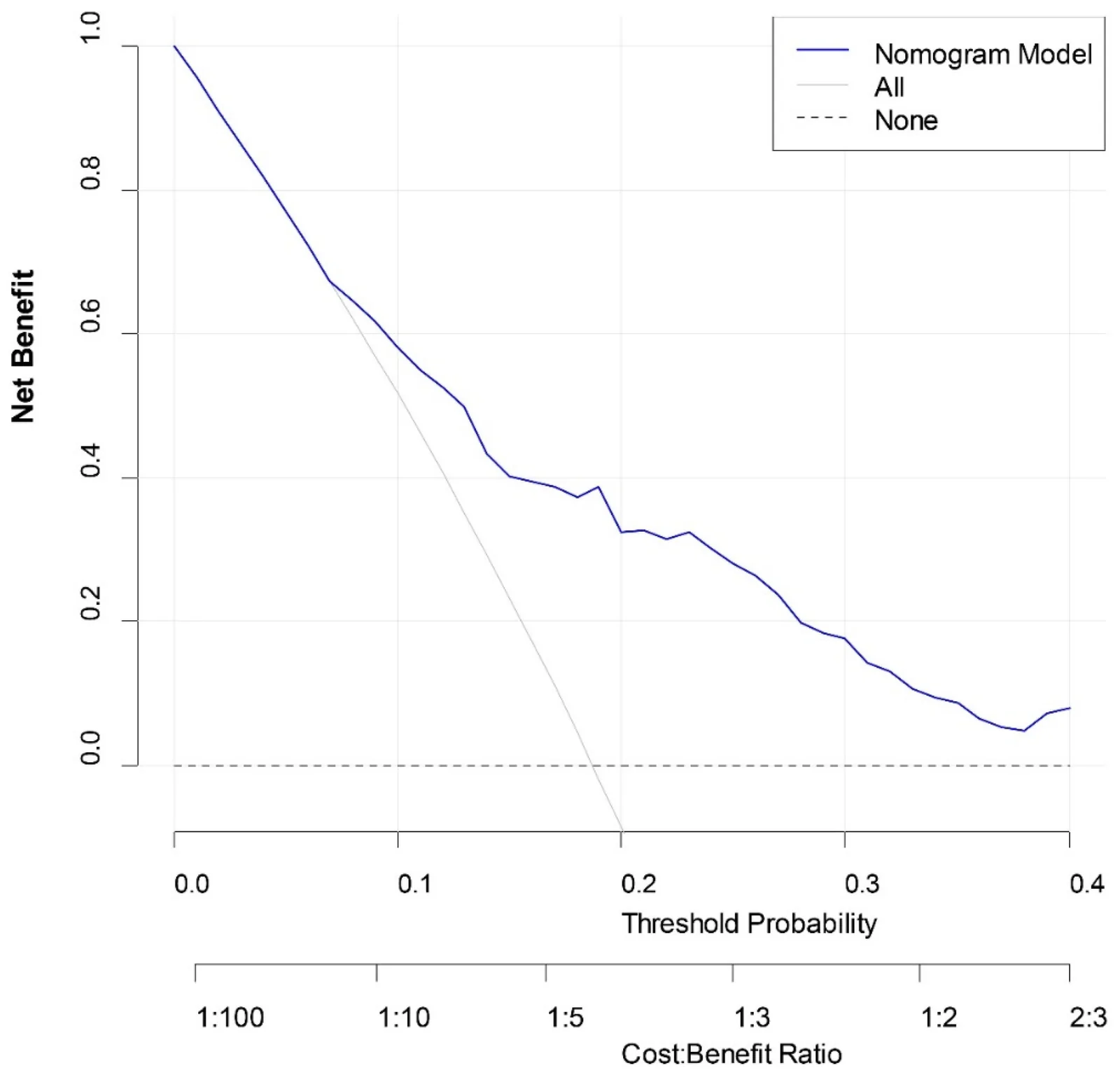
**Decision curve analysis of the nomogram model.** Note: DCA, decision curve analysis. The blue solid line represents the nomogram model; the grey solid line represents the “treat-all” strategy; and the black dashed line represents the “treat-none” strategy. The nomogram model achieved net benefits ranging from 0.079 to 0.956 across threshold probabilities of 0–40%, consistently exceeding the “treat-none” strategy and outperforming the “treat-all” strategy at thresholds ≥ 10% (for example, at 30%: 0.177 vs. −0.863), supporting its clinical utility.

Decision curve analysis (DCA) was performed to evaluate the clinical utility of the nomogram model (Figure 3). Across the threshold probability range of 0–40%, the model demonstrated standardised net benefits ranging from 0.079 to 0.956, consistently exceeding those of the “treat-none” strategy. Compared with the “treat-all” strategy, the model provided greater net benefits at threshold probabilities ≥10%. Within the clinically relevant threshold range of 5–15%, the lower bounds of the 95% confidence intervals for net benefit remained above zero, indicating statistically significant clinical net benefit within this range.

**Figure 4:**
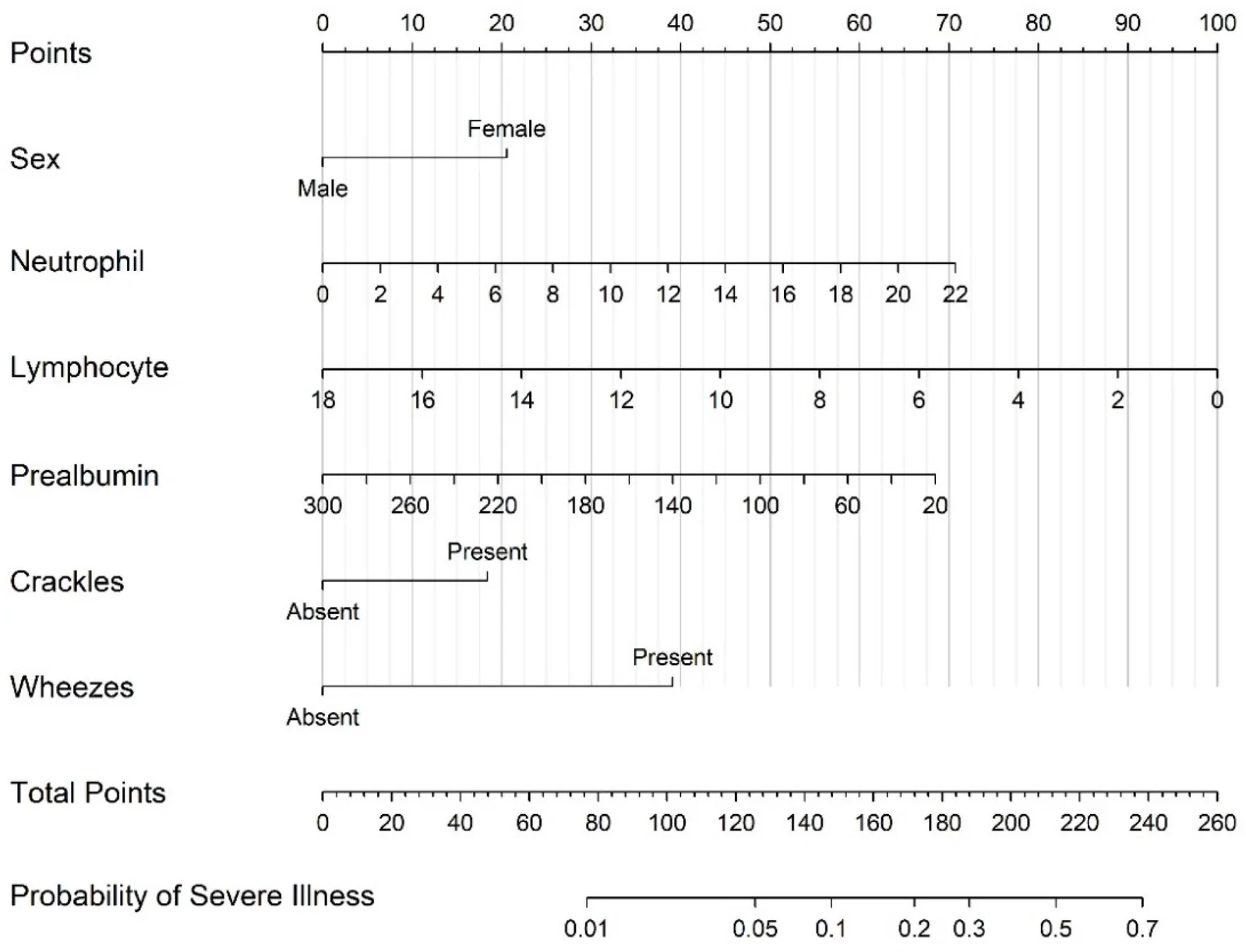
**Nomogram for predicting severe respiratory illness in children with isolated hMPV infection.** Note: The nomogram incorporates six predictors: sex, neutrophil count, lymphocyte count, prealbumin, crackles, and wheezes. The Total Points (range: 0 to 260) are calculated by summing the points assigned to each predictor. Cut-off values of 148 and 188 points correspond to predicted probabilities of 10% and 30%, respectively, defining low-, intermediate-, and high-risk groups. The predicted probability of severe respiratory illness can be obtained from the Risk axis according to the total score.

A nomogram was developed based on the results of the multivariable logistic regression analysis (Figure 4). The total point score ranged from 0 to 260, with predicted probabilities of 10% and 30% corresponding to total scores of 148 and 188, respectively. These cut-off values were used to define low-risk (<10%), intermediate-risk (10–30%), and high-risk (>30%) groups. Severe illness rates were 5.1%, 16.7%, and 46.0% in low-, intermediate-, and high-risk groups, respectively. The high-risk group accounted for 45.5% (46/101) of all severe cases. The trend in severe illness rates across the three risk groups was statistically significant (χ^2^ = 69.105, *p* < 0.001, Table 3).

To assess the potential impact of pre-admission antibiotic exposure on our findings, we reviewed the records of all 1150 children with positive hMPV tests. The rate of documented pre-admission antibiotic use in the isolated hMPV group was 11.85% (64/540). We then performed a sensitivity analysis restricted to the 476 isolated hMPV patients without documented pre-admission antibiotic exposure. In this subgroup, 88 patients (18.5%) developed severe illness, compared with 101/540 (18.7%) in the original cohort. All six predictors retained statistical significance with comparable odds ratios (Table S1). The model achieved an AUC of 0.732 (95% CI: 0.676–0.788) and a Hosmer–Lemeshow *p* value of 0.731, similar to the primary model (AUC = 0.743, 95% CI: 0.690–0.796; HL *p* = 0.139), confirming the robustness of our findings.

## 4. Discussion

After excluding, to the best of our ability, mixed infections and underlying comorbidities, we developed a nomogram incorporating six variables to predict severe respiratory illness in children with isolated hMPV infection. The model demonstrated good discrimination (AUC = 0.743) and calibration performance (Hosmer–Lemeshow test, *p* = 0.139). Among severe cases, 76.2% fulfilled the hypoxaemia/respiratory distress criteria, whereas only 15.8% were classified solely based on radiographic abnormalities, indicating that the model primarily predicts clinical severity characterised by impaired respiratory function. The primary aim of this study was early prediction of subsequent severe respiratory illness using indicators available at admission. To avoid circularity, all predictors were collected at admission, whereas the outcome was determined during follow-up according to the BTS criteria. The BTS criteria do not include any laboratory parameters. The laboratory markers should therefore be understood as early warning signals for subsequent deterioration, rather than as components of the severity definition itself. Furthermore, while crackles and wheezes were more frequent in severe cases, they were also present in 41.91% and 24.15% of non-severe cases, respectively, underscoring that these signs alone are insufficient for risk stratification without multivariable modelling.

Male sex was associated with a lower risk of severe illness compared with female sex (OR = 0.500, 95% CI: 0.311–0.805, *p* = 0.004), which contrasts with most previous studies of paediatric respiratory infections reporting male sex as a risk factor. Williams et al. [11] reported a higher incidence of hMPV lower respiratory tract infection among males, whereas Papenburg et al. [12] observed higher severity scores among girls aged 0–35 months, which is consistent with our findings. Oong et al. [13] found no significant association between sex and disease severity in adults, suggesting that sex-related effects may be age-dependent. Studies involving adults aged ≥50 years have also indicated that severe respiratory syncytial virus (RSV) infection is more strongly associated with male sex, whereas hMPV infection may not exhibit the same sex-specific risk pattern [14]. The moderate effect size of sex observed in our study (OR ≈ 0.5) should therefore be interpreted cautiously and considered together with other clinical factors. The differences between our findings and previous reports may be attributable to differences in study populations, particularly the exclusion of mixed infections and comorbidities. In addition, some studies have suggested that specific viral strains may exhibit greater virulence, although no consensus has been reached [12,15]. Future studies should further validate these findings across different age groups, clinical phenotypes, and viral strains.

Elevated neutrophil count (OR = 1.114, *p* = 0.001) and decreased lymphocyte count (OR = 0.829, *p* = 0.004) were identified as independent predictors of severe illness. hMPV infection promotes neutrophil recruitment through chemokine-mediated pathways [16], and neutrophils can further amplify pulmonary inflammation through the production of reactive oxygen species (ROS) and neutrophil extracellular traps (NETs) [17]. However, neutrophil depletion in mouse models did not improve disease outcomes [18], suggesting that neutrophils may have a dual role during hMPV infection. Regarding lymphocyte responses, CD4+ and CD8+ T cells cooperatively participate in viral clearance, with CD8+ T cells providing protection even in the absence of CD4+ T-cell assistance [19,20,21]. Guo et al. [22] and Yang et al. [23] reported similar immune imbalances in hospitalised children with hMPV infection, and Piñana et al. [24] further demonstrated that lymphopenia was an independent risk factor for lower respiratory tract infection and mortality in immunocompromised populations. These findings suggest that neutrophilia reflects the intensity of the inflammatory response, whereas lymphopenia indicates impaired antiviral immunity, together contributing to the clinical severity spectrum of hMPV infection from both inflammatory and immunological perspectives. Similar immune alterations have also been reported in RSV infection [25], although transcriptomic studies have suggested potential differences between hMPV and RSV in innate immune responses and T-cell-mediated immunity [26].

Prealbumin (transthyretin) was included as a candidate predictor because of its established role as a sensitive negative acute-phase marker, reflecting early inflammatory and nutritional changes during infection. It is a negative acute-phase protein that is more sensitive than albumin to alterations associated with infection, inflammation, and nutritional status [27]. In our study, decreased prealbumin levels were independently associated with severe illness (OR = 0.992, *p* = 0.017). A meta-analysis by Zinellu et al. [28], including 19 studies and 4616 patients with COVID-19, confirmed a significant inverse association between serum prealbumin levels and disease severity and mortality. Similar associations between decreased prealbumin levels and severe COVID-19 have been widely reported [29,30], although evidence remains limited for other common paediatric respiratory pathogens. The persistent independent predictive value of prealbumin after excluding mixed infections and comorbidities in our study suggests its potential utility as a general biomarker for severe viral pneumonia.

Regarding physical examination findings, we included physician-assessed crackles and wheezes rather than caregiver-reported wheezing, as caregivers may misinterpret respiratory sounds caused by nasal congestion, stridor, or airway secretions as wheezing [31]. It is worth noting that, in our cohort, crackles were present in 41.91% of non-severe cases and wheezes in 24.15%, indicating that these signs are neither exclusive to severe illness nor universally present in all hospitalised children. Their independent predictive value was identified through multivariable adjustment rather than from univariate comparisons alone. Such misclassification may overlap with clinical severity criteria. Auscultatory findings obtained by physicians are considered more objective and reproducible. A Slovenian paediatric cohort study demonstrated that wheezes were significantly associated with hypoxaemia in children with hMPV monoinfection, with both bilateral crackles (86% vs. 38%) and wheezes (57% vs. 31%) occurring significantly more frequently in the hypoxaemic group [32]. A German multicentre prospective study also reported higher frequencies of wheezes (63.7% vs. 35.1%) and crackles (64.7% vs. 40.4%) among hospitalised children with hMPV infection compared with outpatients, accompanied by higher proportions of tachypnoea, hypoxaemia, and reduced fluid intake [33]. In our study, both crackles (OR = 1.858, *p* = 0.010) and wheezes (OR = 3.734, *p* < 0.001) were identified as independent risk factors for severe illness, with VIF values of 1.009 and 1.888, respectively, indicating the absence of significant multicollinearity. The particularly strong association observed for wheezes suggests that airway obstruction may represent an important pathway contributing to the progression of severe respiratory illness in children with hMPV infection.

The nomogram demonstrated good gradient discrimination for clinical risk stratification. Notably, only 5.1% of children classified as low risk developed severe illness, suggesting that this threshold may help identify children at low risk among hospitalised patients. The incidence of severe illness in the high-risk group was approximately 2.5 times higher than the overall incidence (18.7%), indicating the need for increased clinical vigilance. The intermediate-risk group comprised 52.2% of all patients (282/540), for whom combined dynamic monitoring, such as changes in oxygenation status and imaging findings, may be warranted. Among children requiring oxygen therapy, the median durations were 2, 3, and 4 days across the three risk groups, showing a stepwise trend consistent with the risk stratification, although the differences did not reach statistical significance (*p* = 0.340), possibly due to limited sample size and individual variability.

Age, CRP, and PCT have been repeatedly identified as risk factors in previous studies investigating hMPV disease severity [7,34]. In our study, CRP (*p* = 0.001) and PCT (*p* = 0.004) levels also differed significantly between the mild and severe groups in univariate analyses; however, neither age nor CRP remained in the final multivariable model. The effect of age may be primarily mediated through lymphocyte count, which reflects immune maturation, whereas CRP may provide overlapping information with neutrophil count, with its independent contribution being attenuated after adjustment for neutrophils. PCT was not included in the regression analysis because of its relatively high proportion of missing data (20.4%). These findings suggest that after excluding comorbidities and mixed infections, the predictive value of conventional inflammatory markers may shift towards indicators that more directly reflect host immune responses.

It is important to emphasise that our model was developed specifically for hospitalised children and is not intended to serve as a screening tool for hospital admission. Rather, its clinical utility lies in early risk stratification after admission: identifying children who, despite being hospitalised, are at substantially higher risk of progressing to severe respiratory illness, thereby enabling tailored monitoring and timely escalation of care. This is particularly relevant given that the majority of hospitalised children (81.3% in our cohort) eventually followed a non-severe course. Several limitations of this study should be acknowledged. First, this was a single-centre retrospective study, and all enrolled children were hospitalised cases. In addition, while clinical assessments were performed under standardised conditions, transient influences such as fever or distress cannot be completely excluded. Therefore, the applicability of the model to other regions, healthcare settings, and non-hospitalised populations requires further evaluation through multicentre prospective studies. Second, the sensitivity of conventional pathogen detection methods is limited, and the possibility of undetected bacterial co-infections cannot be completely excluded. This limitation is particularly relevant given that most specimens were nasopharyngeal swabs, and as reviewed by Gadsby and Musher, obtaining valid lower respiratory tract samples in children remains a major challenge in pneumonia aetiology studies [35]. Furthermore, imaging assessments were based on chest radiography or CT reports without quantitative analysis. Lung ultrasound (LUS) was not used in our cohort. As reviewed by Alexopoulou et al., LUS has been shown to have superior sensitivity for detecting small pleural effusions and can supplement chest radiography in the evaluation of complicated paediatric pneumonia [36]. Therefore, subtle abnormalities such as small-volume effusions might not have been captured, potentially underestimating radiographic severity in a subset of patients. Urine antigen testing for *S. pneumoniae* and *Legionella* was not performed, as these tests were not part of our institutional routine for paediatric CAP.

## 5. Conclusions

In conclusion, we identified six independent predictors and developed a nomogram prediction model with good discrimination (AUC = 0.743) and calibration performance (*p* = 0.139) in 540 children with isolated hMPV infection. Clinical risk stratification based on the model classified children into low-, intermediate-, and high-risk groups, with a significant stepwise increase in severe illness rates (5.1% vs. 16.7% vs. 46.0%, *p* < 0.001). Based on readily available indicators obtained shortly after admission, this model enables early risk stratification in hospitalised children and provides a simple and practical tool for identifying children at risk of severe respiratory illness due to isolated hMPV infection. External validation in multicentre prospective cohorts is warranted.

## Figures and Tables

**Table 1:** Baseline characteristics and laboratory findings of children with non-severe and severe hMPV pneumonia.

	Non-Severe hMPV Group	Severe hMPV Group	*p*
	(n = 439), %	(n = 101), %	
Age	2.85 (0.82–3.92)	3.08 (1.33–4.29)	0.057
Sex (male/female)	250/189	45/56	0.024
WBC, × 10^9/L	7.30 (5.26–10.20)	7.76 (5.49–10.73)	0.010
Neutrophil, × 10^9/L	2.81 (1.66–4.68)	3.60 (2.35–5.72)	0.001
Lymphocyte, × 10^9/L	3.11 (2.06–4.69)	2.52 (1.57–4.14)	0.003
PLT, × 10^9 /L	275.00 (220.00–355.00)	267.00 (220.25–336.25)	0.775
CRP, mg/L	3.66 (0.97–11.75)	7.25 (3.09–17.97)	0.001
ESR *, mm/h	10.50 (6.27–19.00) [n = 322]	13.78 (8.23–25.29) [n = 83]	0.010
PCT *, ng/mL	0.11 (0.07–0.34) [n = 345]	0.21 (0.09–0.60) [n = 85]	0.004
ALT, U/L	17.00 (14.00–23.00)	17.00 (14.00–21.50)	0.627
AST, U/L	46.00 (37.00–61.00)	46.00 (37.00–56.00)	0.778
LDH, U/L	371.00 (300.00–505.00)	380.00 (330.50–508.50)	0.233
Albumin, g/L	41.80 (39.90–44.10)	41.50 (39.15–43.85)	0.272
Prealbumin, mg/L	131.80 (107.50–154.90)	123.80 (100.20–145.20)	0.012
Crackles	184 (41.91)	62 (61.38)	0.001
Wheezes	106 (24.15)	44 (43.56)	<0.001

* ESR and PCT had missing data (25.0% and 20.4%, respectively); medians and comparisons between groups were based on available data.

**Table 2:** Multivariable logistic regression analysis of severe hMPV pneumonia.

	B	S.E.	Wald	df	*p*	Odds Ratio	95% CI Low	95% CI Up
Sex	−0.693	0.243	8.161	1	0.004	0.500	0.311	0.805
Neutrophil	0.108	0.033	10.746	1	0.001	1.114	1.045	1.189
Lymphocyte	−0.187	0.065	8.224	1	0.004	0.829	0.730	0.942
Prealbumin	−0.008	0.003	5.727	1	0.017	0.992	0.985	0.999
Crackles	0.619	0.241	6.623	1	0.010	1.858	1.159	2.977
Wheezes	1.318	0.261	25.544	1	<0.001	3.734	2.240	6.225
Constant	−0.645	0.577	1.246	1	0.264	0.525		

SEX: female as reference (female = 0, male = 1). Neutrophil and Lymphocyte counts are expressed as ×10^9^/L. Prealbumin is expressed in mg/L. Crackles and Wheezes: absent = 0, present = 1. CI: confidence interval; df: degrees of freedom; S.E.: standard error.

**Table 3:** Diagnostic performance of the nomogram-based risk stratification.

Risk Stratum	Predicted Probability	Patients, n	Severe Cases, n	Sensitivity (%)	Specificity (%)	PPV (%)	NPV (%)
Low risk	<10%	158	8	7.9	65.8	5.1	75.7
Intermediate risk	10–30%	282	47	46.5	46.5	16.7	79.1
High risk	>30%	100	46	45.5	87.7	46.0	87.5

Note: PPV, positive predictive value; NPV, negative predictive value. The NPV was calculated using standard diagnostic parameters based on the full cohort.

## Data Availability

The data presented in this study are available on request from the corresponding author due to privacy restrictions.

## Supplementary Materials

The following supporting information can be downloaded at: https://www.mdpi.com/article/10.3390/children13091177/s1, Table S1. Multivariable logistic regression analysis of severe hMPV pneumonia restricted to isolated hMPV patients without pre-admission antibiotic use (n = 476).
