# Supplementary material for: Development and Validation of a Nomogram for Predicting Severe Respiratory Illness in Children with Isolated Human Metapneumovirus Infection
Source: Children (Basel). 2026 Sep 1;13(9):1177. doi: 10.3390/children13091177 (PMC13605185; doi:10.3390/children13091177)
Supplement: Supplementary file 1 [file children-13-01177-s001.zip › children-4513133-supplementary.pdf]

Table S1. Multivariable logistic regression analysis of severe hMPV pneumonia restricted to isolated hMPV patients without pre-admission antibiotic use (n = 476).

	B	S.E.	Wald	df	P	Odds ratio	95% CI Low	95% CI Up
Sex	-0.626	0.257	5.914	1	0.015	0.535	0.323	0.886
Neutrophil	0.078	0.038	4.194	1	0.041	1.081	1.003	1.164
Lymphocyte	-0.219	0.071	9.411	1	0.002	0.803	0.699	0.924
Prealbumin	-0.009	0.004	5.546	1	0.019	0.991	0.984	0.999
Crackles	0.523	0.255	4.223	1	0.040	1.688	1.024	2.781
Wheezes	1.258	0.277	20.611	1	<0.001	3.517	2.043	6.052
Constant	-0.289	0.621	0.216	1	0.642	0.749		

SEX: female as reference (female = 0, male = 1). Neutrophil and lymphocyte counts are expressed as $\times 10^9$/L. Prealbumin is expressed in mg/L. Crackles and wheezes: absent = 0, present = 1. CI: confidence interval; df: degrees of freedom; S.E.: standard error.
